# Cytotoxicity of Printed Aligners: A Systematic Review

**DOI:** 10.3390/dj13070275

**Published:** 2025-06-20

**Authors:** Mauro Lorusso, Fariba Esperouz, Gabriele Di Carlo, Michele Tepedino, Rosa Esposito, Giovanni Pappalettera, Caterina Casavola, Lucio Lo Russo, Domenico Ciavarella

**Affiliations:** 1 Department of Clinical and Experimental Medicine, Dental School of Foggia, University of Foggia, 71122 Foggia, Italy; mauro.lorusso@gmail.com (M.L.); rosa.esposito@unifg.it (R.E.); lucio.lorusso@unifg.it (L.L.R.); domenico.ciavarella@unifg.it (D.C.); 2 Department of Oral and Maxillo-Facial Sciences, Sapienza University of Rome, Viale Regina Elena 287a, 00161 Rome, Italy; gabriele.dicarlo@uniroma1.it; 3 Department of Biotechnological and Applied Clinical Sciences, Dental School of L’Aquila, University of L’Aquila, 67100 L’Aquila, Italy; tepedino.michele@univaq.it; 4 Department of Mechanics, Mathematics, and Management, Polytechnic University of Bari, 70126 Bari, Italy; giovanni.pappalettera@poliba.it (G.P.); caterina.casavola@poliba.it (C.C.)

**Keywords:** cytotoxicity, printed aligners, biocompatibility, critical review

## Abstract

**Background/Objectives:** The capability of printing aligners directly, eliminating the need for a dental model or thermoforming, represents a significant advancement in aligner therapy. This review aimed to assess the cytotoxicity of 3D-printed aligners to clarify their safety profile, given their growing clinical use. **Methods:** An electronic literature search was independently conducted by two reviewers up to February 2025 across PubMed, Scopus, and Web of Science. After a thorough selection process, five in vitro studies were included. The quality and risk of bias were evaluated using the QUIN tool. **Results:** Five studies were included in the systematic review, four of which used TC-85 resin and one TA-28. Two reported no cytotoxic effects. Mild cytotoxicity was observed in one study using UV and heat post-curing, while another reported increased cytotoxicity with extended UV/nitrogen curing. However, notable heterogeneity was present among the studies in terms of the experimental protocols, the cell lines used, and the outcome measures. **Conclusions:** The cytotoxicity of printed aligners appears to be influenced by post-curing duration and system type, highlighting the importance of strict adherence to manufacturers’ protocols. Nevertheless, due to the limited number of studies and methodological variability, definitive conclusions cannot yet be drawn. Further clinical and standardised in vitro studies are needed to better assess the biocompatibility of 3D-printed aligners.

## 1. Introduction

The use of clear aligner therapy has significantly increased due to the growing demand for more aesthetic and less invasive treatment options from patients [1]. The ability to maintain adequate levels of hygiene and comfort has further driven demand for these treatments, particularly among adult patients [2]. A survey conducted in North America found that an increasing number of next-generation orthodontists believe clear aligners will become the primary technique for treating malocclusions [3].

Digital dentistry and the introduction of three-dimensional (3D) printers have revolutionised the field of orthodontics. Charles Hull pioneered the first 3D printing technology in the 1980s, introducing stereolithography, a photopolymerisation process that continues to be widely utilised today [4]. Computer-aided design and the development of new materials have made it possible to manufacture 3D-printed aligners. The ability to directly print aligners without the need for a dental model or thermoforming step marks a significant advancement in aligner treatment. Several types of resins have been proposed for direct printing; however, they were found to be underperforming and were therefore not adopted for aligners. The first resin designed for the direct printing of aligners with a shape memory system was introduced in 2019.

When discussing ‘biocompatibility’, it is important to consider the etymology. The term ‘biocompatibility’ consists of two parts: ‘bio’, meaning life, and ‘compatibility’, meaning ‘able to exist in harmony’ (from the Latin *compatibilis*). As a result, life and compatibility are connected via a close harmony that extends beyond simple compatibility with living organisms; in this context, recent evidence suggests that exposure to micro- and nanoplastics (MNPs) may induce a wide range of adverse effects, including oxidative stress, cytotoxicity, the disruption of internal barriers such as the intestinal lining, the air–blood barrier, and the placental barrier, as well as tissue damage, immune homeostasis imbalance, endocrine disruption, and reproductive and developmental toxicity [5]. These findings underscore the importance of critically assessing the biocompatibility of synthetic materials that may interact with human tissues, particularly in the context of long-term or repeated exposure, such as with 3D-printed orthodontic aligners, differently from the devices used in standard therapeutic approaches [6]. However, it is important to consider that many medical implants labelled as biocompatible are surrounded with a dense, avascular connective capsule that is neither alive nor truly compatible. Consequently, the concept of biocompatibility is often confined to in vitro studies and does not accurately reflect the actual behaviour of a given material. Nonetheless, in vitro cytotoxicity testing remains essential, regardless of the type or duration of contact, to evaluate potential impacts on cellular health. This involves exposing human cell cultures to the product or its derivatives and assessing cell viability through analysis of cell growth, replication, and morphology. Consequently, the concept of biocompatibility is closely associated with another very important concept: cytotoxicity, which refers to the effect of a physical, chemical, or biological agent capable of causing damage to a cell.

Orthodontic treatment with clear aligners typically involves the use of composite resin attachments to facilitate specific programmed aligner movements. The intraoral ageing of orthodontic materials can compromise their structural integrity and affect key material properties, such as hydrolytic stability and plasticisation [7]. These changes may lead to the release of component molecules, including bisphenol-A (BPA), within the oral environment.

Three-dimensional printing technology offers the possibility of planning and manufacturing aligners directly in the office. However, only a few studies [8,9] have analysed the cytotoxicity of the resins used. It is also unclear whether these resins maintain their properties over time. This review was performed to examine the exposure of human cell cultures to 3D-printed resin materials used for clear aligners and analyse their cytotoxic effects.

## 2. Materials and Methods

The current systematic review and meta-analysis were conducted in accordance with the Preferred Reporting Items for Systematic Reviews and Meta-Analyses (PRISMA) standards [10]. The protocol was submitted and registered in the PROSPERO database: CRD42025641592.

### 2.1. Search Strategy and Database Screening

An electronic search of the English-language literature was conducted up to February 2025 using the PubMed, Scopus, and Web of Science databases. In addition, a manual search was performed using the bibliographies of all reviewed articles. The research process was conducted using a combination of MeSH terms and free-text words, combined with Boolean operators (AND, OR). The following search string was used for PubMed, Scopus, and Web of Science: (biocompatibility) AND (cytotoxicity) AND (3D printed aligners).

### 2.2. Eligibility Criteria

#### 2.2.1. Inclusion Criteria

No publication year restrictions were applied to the search. Studies were screened based on the following inclusion criteria: publications in English; cohort, case-control, observational and retrospective, or longitudinal studies; and in vitro studies investigating the cytotoxicity of 3D-printed aligner materials on human gingival cell lines.

#### 2.2.2. Exclusion Criteria

The exclusion criteria were systematic reviews and meta-analyses, as well as studies not published in English.

### 2.3. Focused PICO Question and Effect Measure

The study was structured according to the PICO framework:

- Population (P): 3D-printed resin materials used for clear aligners.
- Intervention (I): Exposure to human cell cultures.
- Comparison (C): Other dental materials or no exposure.
- Outcome (O): Cytotoxic effects, including cell viability, apoptosis, and inflammatory response.

### 2.4. Study Screening and Inclusion

After all information was gathered from the computer search, duplicates were removed. A screening process was then conducted independently by two authors (F.E. and M.L.) using the list of publication titles obtained from the combined database searches. Titles deemed eligible were further assessed by reviewing their abstracts, eliminating articles that did not meet the inclusion criteria. The full texts of the selected abstracts were then retrieved and evaluated based on the predefined criteria, resulting in a final list of selected publications. To ensure the relevance of the included studies, both examiners independently verified the articles. In cases of disagreement, a third author (M.T.) was consulted to make the final decision.

### 2.5. Data Extraction

Data extraction was carried out independently by two reviewers (F.E. and M.L.), with results compared and merged using an ad hoc extraction sheet. The articles were jointly re-evaluated with the third reviewer (M.T.) to reach a consensus on the inclusion of studies and relevant variables. The extraction sheet, formatted in Excel, included the following fields: author names, year of publication, country, study design, materials, cell line, positive control, post-curing process, analysis, and results (Table 1).

### 2.6. Assessment of Risk of Bias

The quality assessment and risk of bias of the included studies were evaluated using the Quality Assessment Tool for In Vitro Studies (QUIN tool) [14], which assesses the risk of bias in in vitro research. Each study was scored according to the 12 domains, with a maximum score of 24. Based on the total score, studies were classified as follows: low risk of bias (≥20), moderate risk (15–19), and high risk (≤14), in accordance with published recommendations. Two authors (F.E. and M.L.) independently conducted the assessment, and any discrepancies were resolved in a joint meeting with the third reviewer (M.T.), who also independently repeated the risk of bias assessment and assigned a score according to the QUIN tool.

## 3. Results

### 3.1. Study Selection

**Figure 1:**
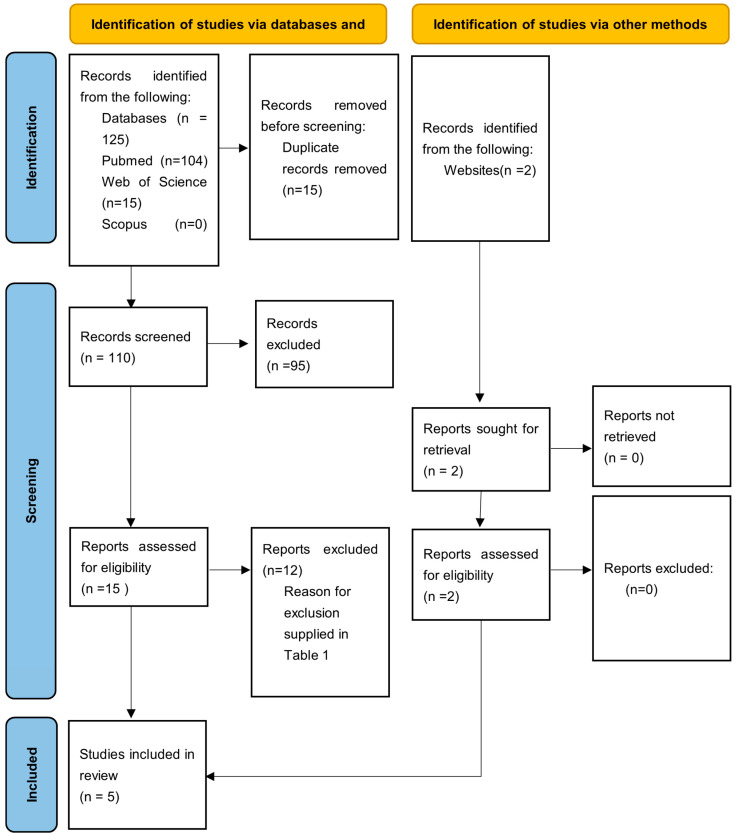
PRISMA flow diagram of the article selection process.

The electronic search retrieved a total of 125 articles from database searches and 2 additional articles from a hand search, covering publications up to January 2025. After removing 15 duplicates, title and abstract screening was conducted on 110 articles, of which 95 were excluded based on relevance. A full-text analysis was then performed on the remaining 16 articles, leading to the exclusion of 12 more studies. The final review included five articles [8,9,11,12,13] (Figure 1).

### 3.2. Risk of Bias Assessment

**Figure 2:**
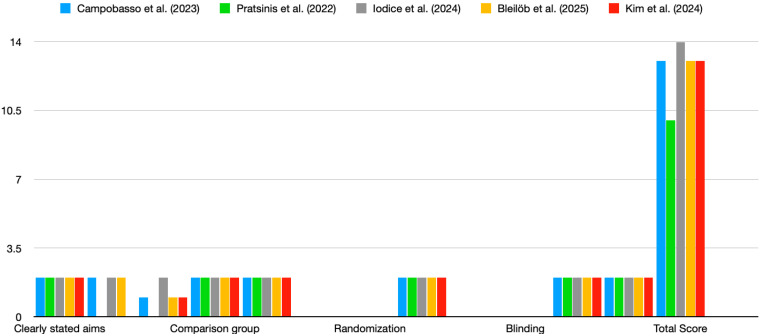
Risk of bias. Scores for studies were assigned as follows: adequately specified = 2; inadequately specified = 1; not specified = 0. Color coding: blue—Campobasso et al., (2023); green—Pratisinis et al., (2022); grey—Iodice et al., (2024); yellow—Bleilob et al., (2025); red—Kim et al., (2025). [8,9,11,12,13].

The studies reviewed exhibited varying levels of risk of bias based on the QUIN tool evaluation [14]. Most studies did not fully address criteria related to randomisation, blinding, and operator details, which may have affected the overall reliability of their findings (Figure 2). The four studies included in the review demonstrated a moderate risk of bias, with a QUIN tool score of <70%. Figure 2 highlights consistent strengths across studies in terms of clearly stated aims, adequate methodology, and statistical analysis. However, major weaknesses include the lack of blinding, randomisation, and operator details. These gaps suggest a need for more rigorous and standardised reporting to minimise bias in future in vitro studies on the cytotoxicity of 3D-printed aligners.

### 3.3. Study Characteristics

The eligible studies (Table 1) were conducted between 2022 and 2024 in various countries, including Germany [13] Greece [8], Italy [9,11], and Republic of Korea [12]. All five studies were in vitro investigations testing Tera Harz TC85 (Graphy, Seoul, Republic of Korea), with one study also analysing Form Cure (Formlabs, Somerville, MA, USA) [9] and Tera Harz TC28 (Graphy, Seoul, Republic of Korea) [13] Tera Harz Cure (THC 2). Aligners were cured for 14 min using a nitrogen generator curing machine, whereas Form Cure aligners were post-cured for 30 min on each side (total: 60 min) at 405 nm UV LED. In the studies by Kim et al. [12] and Iodice et al. [11], aligners made from Tera Harz TC85 were cured using the THC 2 (Graphy) for 20 min under a nitrogen atmosphere. In the study by Pratsinis et al. [8], the post-curing process was performed using a Cure M curing machine (Graphy) with 24 min of UV curing on both the internal and external sides of the aligners. In the study by Bleilöb et al. [13], the post-curing process was carried out using the Tera Harz Cure THC 2 (Graphy Inc., Seoul, Republic of Korea) under a 95% nitrogen atmosphere. Specimens were exposed to UV light for 20, 30, or 60 min at curing level 2, followed by a 2 min wash in boiling water.

The included studies reported different outcomes: Iodice et al. [11] examined the effects of post-curing on cytotoxicity, Kim et al. [12] investigated the impact of temperature and centrifugation time on the removal of uncured resin, Campobasso et al. [9] assessed cytotoxicity in different post-curing methods, and Pratsinis et al. [8] evaluated both the cytotoxicity and oestrogenicity of printed aligners. Various culture media were used, including RPMI 1640 and Dulbecco’s Modified Eagle Medium, along with different cell lines, such as pre-osteoblast mouse calvaria and human gingival fibroblasts. Incubation times also varied significantly, ranging from 4 to 24 h and up to 14 days. Because of the substantial variability among the studies, a meta-analysis could not be conducted.

## 4. Discussion

This review highlights the cytotoxicity of printed aligners, which represents a crucial aspect of orthodontic practice. Despite their use for several years, only a few studies have examined this critical issue. The analysed studies focused on the use of a specific light-cured polyurethane resin, TC-85, produced by Graphy (Seoul, Republic of Korea).

The evaluation of medical device biocompatibility is guided by the ISO 10993 series of international standards [15]. These standards outline specific requirements based on the device type and its intended use, with various tests detailed in the standard’s subchapters to ensure biosafety [16]. For instance, devices that come into contact with circulating blood require an assessment of hemocompatibility (ISO 10993-4) [17], along with other relevant endpoints. For long-term use, chronic toxicity evaluations (ISO 10993-11) [18] are also mandatory. In line with ISO 10993-2, in vivo testing should be minimised to reduce reliance on animal studies [19].

Cytotoxicity refers to the effect of a physical, chemical, or biological agent capable of causing damage to a cell. ISO 10993-5 outlines the specifications for in vitro cytotoxicity testing [20]. To accommodate the broad spectrum of medical devices, the standard is deliberately flexible in several aspects. This flexibility allows test designs to be customised to meet the specific requirements of clinical applications [21]. Additionally, the adaptable nature of these specifications permits modifications to laboratory protocols in response to technological advancements, resulting in a wide range of available test designs.

After the printing phase, manually removing print media and excess resin is essential to ensure aligner safety through post-curing [22]. This step is crucial because the surfaces of 3D-printed devices often contain residual uncured resin immediately after printing. Most manufacturers recommend using an activated bath of organic solvents, such as ethanol or isopropyl alcohol (IPA), to clean the surfaces of 3D-printed devices [23]. Although opinions vary regarding the optimal IPA washing time, Jang et al. [22] suggested that soaking in IPA for 1 min is clinically effective. However, the chemical effects of these solvents on acrylic-based polymers can reduce the translucency of clear resin materials [24]. Additionally, organic solvents may cause unpredictable alterations to the surface’s physical properties. For this reason, centrifugation has been proposed as the safest cleaning method for 3D-printed transparent aligners.

The step following the removal of excess resin is post-polymerisation, a process in which the printed material undergoes curing after 3D printing. This step is essential to ensure the mechanical properties of the aligner and to enhance the biocompatibility of 3D printing resins [25,26]. Different post-polymerisation procedures can influence cytotoxicity levels because the degree of conversion of the 3D photopolymer material directly affects the resin’s biocompatibility [26].

In the study by Pratsinis et al. [8], 10 sets of aligners were soaked in sterile deionised water for 14 days, and the cytotoxicity and oestrogenicity of the released factors were evaluated using MTT (3-[4,5-dimethylthiazol-2-yl]-2,5-diphenyltetrazolium bromide) assays. These assays were conducted on human gingival fibroblasts, as well as on oestrogen-sensitive MCF-7 and oestrogen-insensitive MDA-MB-231 breast cancer cell lines. Additionally, 17β-oestradiol and bisphenol A were used as positive controls. The aligners were cured for 24 min on both the inner and outer sides using Cure M (Graphy, Seoul, Republic of Korea). The results of the study indicated that, during the 14-day ageing period, the released factors were not cytotoxic to human gingival fibroblasts and did not affect intracellular reactive oxygen species levels. Furthermore, no oestrogenic effects were observed.

In the study by Campobasso et al. [9], the cytotoxicity of printed aligners was analysed under different post-polymerisation conditions. The aligners were printed using the same 3D printing resin (TC-85DAC; Graphy, Seoul, Republic of Korea) and printer (AccuFab-L4D; Shining 3D Tech. Co., Hangzhou, China). For post-curing, six aligners were treated for 14 min using the Tera Harz Cure and a nitrogen generator curing machine (THC2; Graphy). The other six aligners underwent post-curing for 30 min on each side using the Form Cure machine (Formlabs). The results showed that 3D-printed aligners post-cured using the Tera Harz system with a nitrogen generator were biocompatible. By contrast, aligners post-cured using the Form Cure system exhibited mild cytotoxicity. According to Ahrari et al. [27], cell survival ranges between 60% and 90% in cases of mild cytotoxicity.

In the study by Iodice et al. [11], the cytotoxicity of printed aligners was analysed using three different post-curing durations (14, 24, and 50 min). The samples were incubated in saliva for 14 days, after which the supernatant was collected. To assess potential cytotoxicity, human gingival fibroblasts (HGF-1 and CRL-2014) were used. Additionally, HGF-1 cells were seeded onto the specimens and a glass control sample, with cell viability evaluated after 72 h of incubation. The results showed that directly 3D-printed aligners exhibited a cytotoxic effect on fibroblast growth comparable to that of conventional thermoformed aligners. Furthermore, a negative linear trend was observed between curing time and fibroblast growth, indicating that longer polymerisation times were associated with greater cytotoxic effects.

Kim et al. [12] investigated the impact of temperature and centrifugation time on the removal of uncured resin from 3D-printed clear aligners. Aligners were cleaned using IPA or centrifugation (gravitational acceleration: 27.95 g) under two temperature conditions: room temperature (23 °C) and high temperature (55 °C), for durations of 2, 4, and 6 min. The cleaning efficiency of each method was evaluated using rheological analysis, weight measurements, transparency assessments, scanning electron microscopy, 3D geometry evaluation, stress relaxation testing, and cell viability testing. The findings revealed that higher temperatures and longer centrifugation times significantly decreased aligner viscosity, weight (*p* < 0.05), and transmittance. Aligners cleaned with IPA demonstrated noticeably lower transparency and rougher surfaces as confirmed via scanning electron microscopy. Despite these variations, all treatment groups met ISO biocompatibility standards in cytotoxicity tests. Furthermore, stress relaxation testing showed that all aligners achieved > 95% recovery within 60 min.

The study of Bleilöb et al. [13] demonstrated that the cell viability of human gingival fibroblasts decreased with increasing material thickness, particularly at 2 mm and above, when specimens were post-cured for the manufacturer-recommended 20 min. Extending the UV post-curing duration to 30 or 60 min did not result in improved biocompatibility. Importantly, none of the tested conditions led to a reduction in cell viability greater than 30%, thereby meeting the ISO 10993-5 [20,28] threshold for non-cytotoxicity. Additionally, exposure to saliva—both conditioned and unconditioned—significantly reduced cell proliferation, highlighting the potential impact of the intraoral environment on aligner material performance.

The analysed studies revealed significant differences, particularly in cytotoxicity, which showed a strong correlation with the post-curing machine used. Notably, among the studies reviewed, only three [9,11,12] utilised the same post-curing machine, Tera Harz Cure (Graphy), which employs a combined system of UV energy and nitrogen irradiance. The other post-curing machines, Form Cure (Formlabs) and Cure M (Graphy), relied solely on UV systems. Additionally, post-curing times varied, ranging from 14 to 50 min, which plays a critical role in the biological safety of aligners. As highlighted by Iodice et al. [11], longer post-curing durations are associated with increased cytotoxicity. Among the analysed studies, only three did not demonstrate cytotoxicity [8,12,13]. It is worth noting that, in the study by Campobasso et al. [9], mild cytotoxicity was observed only in the group of aligners post-cured with a UV-only system. Therefore, the post-curing method employed may influence cytotoxicity and, consequently, the safety profile of printed aligners. Finally, Iodice et al. [11] demonstrated that printed aligners exhibit a cytotoxic effect comparable to that of thermoformed aligners. Furthermore, the authors observed a linear relationship between polymerisation time and cell growth, suggesting that printed aligners may exhibit greater cytotoxicity with longer polymerisation times.

## 5. Limitations

The limitations of this review stem primarily from the small number of included studies and the absence of randomised clinical trials. In addition, the available in vitro studies showed considerable heterogeneity in terms of study protocols, cell lines, post-curing procedures, and outcome measures, which makes it difficult to draw definitive conclusions or compare results across studies. Consequently, the findings should be interpreted with caution and considered preliminary. Future research should aim to adopt standardised and reproducible in vitro methodologies and include well-designed clinical investigations, as the safety and biocompatibility of 3D-printed aligners may have significant implications for patient health.

## 6. Conclusions

This critical review has highlighted two key factors related to the cytotoxicity of printed aligners: post-curing time and the type of device used for post-curing. The findings suggest that post-curing with UV-only technology may be associated with mild cytotoxic effects, while longer post-curing durations could increase the risk of cytotoxicity. Therefore, it is essential to adhere to manufacturers’ specified curing times and follow a strict manufacturing process to ensure the biocompatibility and safety of printed aligners.

For this reason, the use of these materials requires meticulous attention at every stage of production. As their development continues to advance, it becomes increasingly important to gain a comprehensive understanding not only of their advantages and limitations but especially of their health and safety profiles. Consequently, it is recommended that future in vivo studies investigate the effects of deviations from the manufacturers’ recommended timescales in order to more accurately assess the potential health impacts of these resins.

## Figures and Tables

**Table 1:** Characteristic of included studies.

ID	Year	Country	Study Design	Materials	Post-Curing Process	Cell Line	Positive Control	Analysis	Results
Pratsinis et al. [8]	2022	Greece	Study in vitro	Tera Harz TC85 A aligner resin (Graphy, Seoul, Republic of Korea)	Cure M curing machine (Graphy, Seoul, Republic of Korea) 24 min of UV curing on both internal and external aligner sides	Gingival fibroblast	17b-Estradiol (E2; 10-9 M) and BPA (10-8 M) were used as positive controls	In sterile deionised water for 14 days, the final numbers of viable cells were estimated by a modification of the MTT method	No signs of cytotoxicity were seen for the aligner samples for concentrations (*v*/*v*) of 20% (P5 0.32), 10% (P5 0.79), or 5%(P5 0.76)
Iodice et al. [11]	2024	Italy	Study in vitro	Tera Harz TC- 85 DAC resin	Tera Harz Cure (Graphy, Seoul, Republic of Korea) Curing times: 14, 24, and 50 min under high UV energy and nitrogen irradiance	human gingival fibroblasts (HGF-1)-CRL2014	Zendura FLX material, were then placed in a separate plastic tube each, containing 2.5 mL of centrifuged saliva	37 °C in a water bath for 14 days and centrifugation the collected supernatant was filtered using a 0.22 mm pore-size filter were stored at −80 °C	The 3D-printed aligner sample with centrifuged saliva significantly reduced human fibroblasts’ viability; interestingly, pure saliva (saliva without aligners) also reduced cell viability, suggesting that salivary enzymes may influence cell viability
Campobasso et al. [9]	2023	Italy	Study in vitro	Tera Harz TC-85 DAC resin and Form Cure	THC2 (Graphy, Seoul, Republic of Korea): aligners were cured for 14 min using a nitrogen generator curing machine (P1); Form Cure (FormLabs, Somerville, MA, USA): aligners were post-cured for 30 min on each side (total: 60 min) at 405 nm UV LED (P2).	Pre-osteoblast mouse calvaria MC3T3E-1 cells	The DMEM medium with cultured cells, without any aligner specimens	The cultures were washed with PBS-EDTA, the cells treated with an MTT solution (diluted 1:10 in DMEM) and incubated for 4 h, following which the formazan was solubilised using 100% DMSO	Form Cure procedures reported significantly moderate cytotoxicity
Kim et al. [12]	2024	Republic of Korea	Study in vitro	Tera Harz TC-85	Tera Harz Cure THC 2 UV curing system for 20 min curing process under nitrogen atmosphere	Murine fibroblast cell line	NI	MTT method	No cytossicity
Bleilöb et al. [13]	2025	Germany	Study in vitro	Tera Harz TC-28	Specimens were post-cured for 20, 30, or 60 min using the Graphy Cure THC 2 unit in a 95% nitrogen atmosphere	Human gingival fibroblasts (HGFs)	NI	37 °C for 12 days in culture medium or saliva Then, transferred to HGF-seeded 96-well plates Proliferation monitored via live-cell imaging (Incucyte) Viability measured after 48–72 h with AlamarBlue assay	No cytossicity

## Data Availability

No new data were created or analyzed in this study. Data sharing is not applicable to this article.
